# Molecular Crystal Structure Simulations and Structure-Magnetic Properties of LiFePO_4_ Composite Particles Optimized by La

**DOI:** 10.3390/molecules29163933

**Published:** 2024-08-20

**Authors:** Qing Lin, Kaimin Su, Yajun Huang, Yun He, Jianbiao Zhang, Xingxing Yang, Huiren Xu

**Affiliations:** 1 College of Biomedical Information and Engineering, Hainan Medical University, Haikou 571199, China; 2 Guangxi Key Laboratory of Nuclear Physics and Nuclear Technology, College of Physics and Technology, Guangxi Normal University, Guilin 541004, China; 3 Department of Civil Engineering, Jiangxi Water Resources Institute, Nanchang 330013, China

**Keywords:** LiFePO_4_, composite, crystal structure, magnetic, cathode material, Mössbauer

## Abstract

In this study LiFePO_4_/C composite particles were synthesized using five different carbon sources via a one-step sol-gel method. La-doped LiFePO_4_ was also synthesized using the sol-gel method. The XRD pattern of Li*_x_*La*_y_*FePO_4_ (*x* = 0.9~1.0, *y* = 0~0.1) after being calcined at 700 °C for 10 h indicates that as the doping ratio increased, the sample’s cell volume first increased then decreased, reaching a maximum value of 293.36 Å^3^ (*x* = 0.94, *y* = 0.06). The XRD patterns of Li_0.92_La_0.08_FePO_4_ after being calcined at different temperatures for 10 h indicate that with increasing calcination temperature, the (311) diffraction peak drifted toward a smaller diffraction angle. Similarly, the XRD patterns of Li_0.92_La_0.08_FePO_4_ after being calcined at 700 °C for different durations indicate that with increasing calcination times, the (311) diffraction peak drifted toward a larger diffraction angle. The infrared spectrum pattern of Li*_x_*La*_y_*FePO_4_ (*x* = 0.9~1.0, *y* = 0~0.1) after being calcined at 700 °C for 10 h shows absorption peaks corresponding to the vibrations of the Li–O bond and PO_4_^3-^ group. An SEM analysis of Li*_x_*La*_y_*FePO_4_ (*x* = 1, *y* = 0; *x* = 0.96, *y* = 0.04; *x* = 0.92, *y* = 0.08) after being calcined at 700 °C for 10 h indicates that the particles were irregular in shape and of uniform size. The hysteresis loops of Li_0.92_La_0.08_FePO_4_ after being calcined at 600 °C, 700 °C, or 800 °C for 10 h indicate that with increasing calcination temperature, the Ms gradually increased, while the Mr and Hc decreased, with minimum values of 0.08 emu/g and 58.21 Oe, respectively. The Mössbauer spectra of Li*_x_*La*_y_*FePO_4_ (*x* = 1, *y* = 0; *x* = 0.96, *y* = 0.04; *x* = 0.92, *y* = 0.08) after being calcined at 700 °C for 10 h indicate that all samples contained Doublet(1) and Doublet(2) peaks, dominated by Fe^2+^ compounds. The proportions of Fe^2+^ were 85.5% (*x* = 1, *y* = 0), 89.9% (*x* = 0.96, *y* = 0.04), and 96.0% (*x* = 0.92, *y* = 0.08). The maximum IS and QS of Doublet(1) for the three samples were 1.224 mm/s and 2.956 mm/s, respectively.

## 1. Introduction

**Figure 1:**
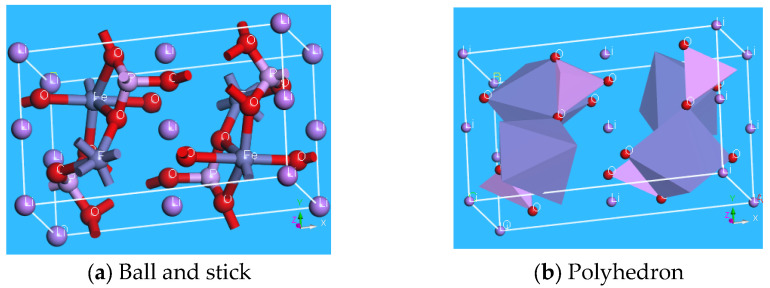
Crystal structure of LiFePO_4_.

Compared with traditional rechargeable secondary batteries, lithium-ion batteries, as a new type of rechargeable battery, offer numerous advantages, including a high voltage, long cycle life, and environmental friendliness. The primary cathode materials for lithium-ion batteries include lithium cobalt oxide (LiCoO_2_), lithium nickel oxide (LiNiO_2_), lithium manganese oxide (LiMn_2_O_4_), lithium manganese dioxide (LiMnO_2_), and lithium iron phosphate (LiFePO_4_). In 1997, A.K. Padhi, K.S. Nanjundaswamy, and J.B. Goodenough proposed olivine-type LiFePO_4_ as a new cathode material [1]. The crystal structure of LiFePO_4_ is shown in Figure 1. Olivine-type LiFePO_4_, used as a cathode material in lithium-ion batteries, features a slightly distorted, hexagonal, close-packed arrangement of internal atoms. It belongs to the orthorhombic crystal system with a space group of Pnma [2], in which phosphorus (P) atoms are bonded to four oxygen (O) atoms to form the PO_4_ structure. Lithium (Li) and iron (Fe) atoms form octahedral structures, LiO_6_ and FeO_6_, respectively, with six surrounding O atoms. The magnetic properties of LiFePO_4_ are primarily antiferromagnetic below the Neél temperature *T*_N_, due to superexchange interactions between the internal Fe atoms through the Fe–O–P–O–Fe bond [3].

LiFePO_4_ offers several advantages, including inexpensive raw materials, a high theoretical capacity [4,5], high energy density, good stability, and non-toxicity [6,7]. However, its application as a cathode material is mainly limited by its low conductivity [8,9]. There are three primary methods to improve conductivity: reducing grain size, doping with other metal ions, and coating with conductive elements [10,11]. Guangcong Zeng et al. [12] prepared both coated and uncoated LiFePO_4_ materials and found that the sample that was coated with C-Nb_2_CTx exhibited superior electrochemical properties. Similarly, Xiaohua Chen et al. [13] prepared zinc oxide and carbon co-modified LiFePO_4_ nanoparticles (LFP/C-ZnO) using an inorganic-based hydrothermal route and found this to significantly boost its performance. Meanwhile, Abdurrahman Yolun et al. [14] prepared an Ru-substituted LiFePO_4_ cathode material and demonstrated that it exhibits excellent electrochemical performance. There are many synthetic methods for producing olivine-type LiFePO_4_, including the sol-gel method [15,16], hydrothermal method [17,18], co-precipitation method [19,20], high-temperature solid-state reaction method [21,22], microwave method [23,24], and thermal reduction method [24,25,26]. The sol-gel method, in particular, is an important approach for preparing LiFePO_4_; due to its homogeneous precursor, low heat treatment temperature, simplicity of equipment, and ease of control, this method is widely favored among researchers [27].

Furthermore, Dan Li et al. [28] prepared carbon-coated Li_1-3_*_x_*La*_x_*FePO_4_/C (*x* = 0~0.025) materials with smaller particles and uniform morphology by combining solid state reaction and microwave heating. The results showed that the conductivity was improved after La-ions doping, thereby increasing the discharge capacity of the electrode material. Yung Da Cho et al. [29] prepared La-doped LiFePO_4_ materials, and the results showed that the structure remained unchanged after La-ion doping. However, the cyclic stability of the electrode materials’ capacity was effectively improved. Shaohua Luo et al. [30] prepared Li_1-*x*_La*_x_*FePO_4_ (*x* = 0.0025~0.01) through a two-step solid state reaction. They found that the microstructure and grain size of samples doped with La ions hardly changed, and, among the samples, Li_0.99_La_0.01_FePO_4_ showed the most excellent electrochemical performance. The carbon source also has a significant influence on the performance of LiFePO_4_ [31]. Chaoqi Shen et al. [32] prepared high-performance LiFePO_4_/C composite using an optimized solid-state synthesis route. M. Swierczynski et al. [33] found that lithium iron phosphate carbon (LiFePO_4_/C) composite demonstrates excellent performance, with 8000 complete service cycles at 25 °C. Xingling Lei et al. [34] prepared LiFePO_4_ cathode material by introducing carbon and found that it exhibits good crystallinity and is of the olivine type, with a microscopic particle size of approximately 200 to 500 nm. Seo Hee Ju et al. [35] observed that pure olivine can be prepared by adding carbon, and increasing the amount of nano carbon black will, to a certain extent, increase the particle size of LiFePO_4_.

In this study, we systematically investigated the effects of different carbon sources and La doping on the structure and properties of LiFePO_4_. Five different carbon sources, including ethylene glycol (C_2_H_6_O_2_, analytical grade AR), polyethylene glycol 4000 (PEG4000; HO(CH_2_CH_2_O)*_n_*H, chemically pure CP), polyvinyl alcohol (PVA-124; [CH_2_CH(OH)]*_n_*, analytical grade AR), citric acid (C_6_H_8_O_7_·H_2_O, analytical grade AR), and glucose (C_6_H_12_O_6_·H_2_O, analytical grade AR), were used, and LiFePO_4_/C composite particles were synthesized using a one-step sol-gel method. Then, Li*_x_*La*_y_*FePO_4_ (*x* = 0.9~1.0, *y* = 0~0.1) materials were prepared using the sol-gel method, which offers precise control, straightforward operation, and simple synthesis conditions. The magnetic properties of the samples with varying doping ratios, calcination temperatures, and calcination times were studied to select the optimal doping ratio, calcination temperature, and calcination time, thereby improving the properties of the samples for an improved application value.

## 2. Results and Discussion

### 2.1. XRD Analysis

**Figure 2:**
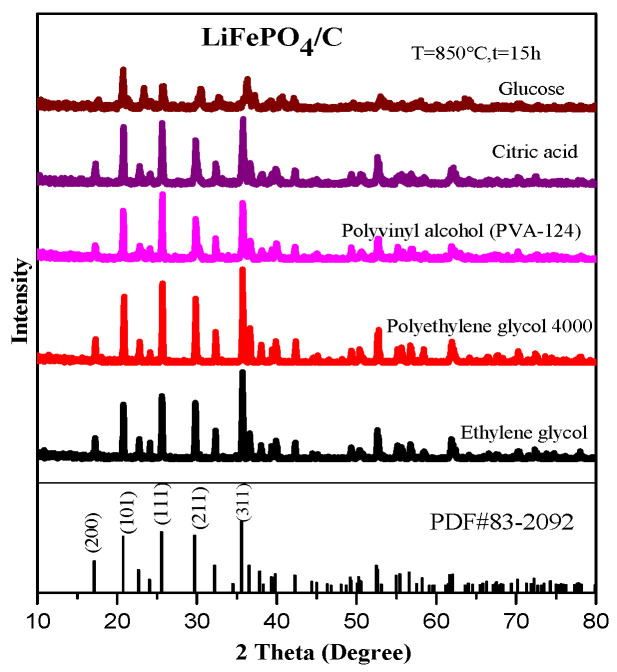
XRD diagram of LiFePO_4_/C composite.

To study the phase structure and average lattice parameters of the prepared LiFePO_4_/C composite particles, a crystal X-ray diffraction (XRD) analysis was conducted using an X-ray diffractometer. The obtained XRD data were analyzed using MDI Jade 6 software, and it was found that its results corresponded most closely with the standard card PDF#83-2092. Figure 2 presents the XRD diffraction patterns of the LiFePO_4_/C composites that were synthesized from five different carbon sources. The main diffraction peaks (200), (101), (111), (211), and (311) of each composite align precisely with those in the standard card PDF#83-2092, indicating that each composite sample has a complete pure phase LiFePO_4_ peak type, belonging to the space group Pnma [36], without any detected impurity peaks and demonstrating good crystallinity. The uneven baseline of the diffraction pattern may be attributed to the presence of amorphous carbon, which is consistent with the XRD diffraction patterns of the LiFePO_4_/C composites prepared by C. Chen et al. [37], Xiaozhen Liao et al. [38], and Jae Kwang Kim et al. [39]. In these studies, the diffraction patterns also displayed complete LiFePO_4_ peaks, but with a similarly uneven baseline.

Among the peaks, the main peak (311) of the XRD diffraction pattern of the sample using PEG4000 as the carbon source is notably sharp, which is similar to the XRD diffraction pattern reported by Zhihui Xu et al. [40] for the LiFePO_4_/C that was prepared with PEG4000. In contrast, the XRD diffraction peaks of some samples within the prepared materials are less distinct and not particularly sharp, which resembles the X-ray diffraction pattern of the composite material that was prepared using glucose as the carbon source. They believe that this phenomenon may be caused by amorphous or low crystalline carbon. The XRD diffraction pattern of the LiFePO_4_/C that was prepared with ethylene glycol as a carbon source is similar to the pattern reported by C. Guan et al. [41], who used ethylene glycol as an additive in the sol-gel method. The diffraction peak at the position of the corresponding size 2θ is sharp and distinct, exhibiting a complete LiFePO_4_ peak profile, and the relative intensity of the peak corresponds to the standard card.

### 2.2. Preparation Process 

The average lattice constants of the LiFePO_4_/C composite samples that were synthesized from different carbon sources are shown in Table 1. It shows that the average lattice constants a-axis and b-axis of the composites are smaller than those of the standard pure phase LiFePO_4_ (PDF#83-2092), which aligns with the findings of Dragana Jugovića et al. [42] and Xiaodong Wang et al. [43], who prepared LiFePO_4_/C composites with similar lattice parameters. Similar to the approach used by Huisheng Huang et al. [44], who utilized Materials Studio 4.0 and other software to simulate LiFePO_4_/C, this study also employs these tools to understand the band gap and density of states of the energy band structure of the LiFePO_4_ composite. The average lattice parameters were obtained through XRD testing, as well as the file 2,100,916.

**Figure 3:**
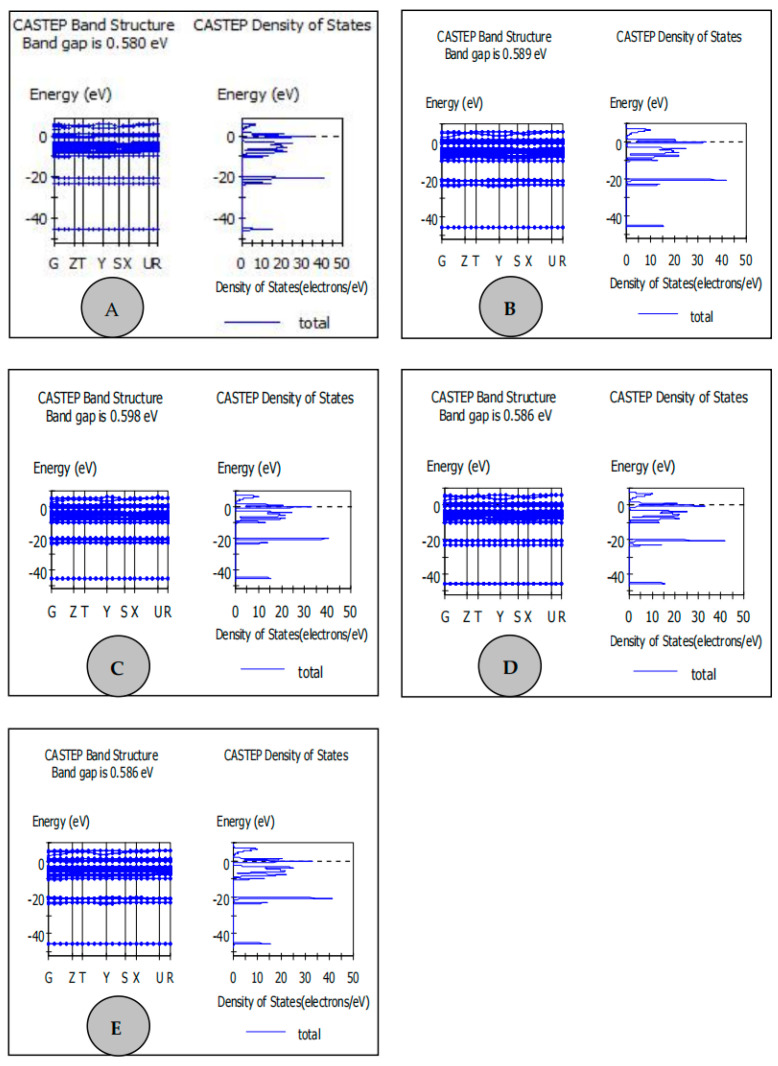
Energy band structure, band gap, and density of states of LiFePO_4_ composites. (Different carbon sources: (**A**) Ethylene glycol; (**B**) Polyethylene glycol 4000; (**C**) Polyvinyl alcohol PVA-124; (**D**) Citric acid; (**E**) Glucose).

The band gap and density of states of the energy band structure for the prepared LiFePO_4_/C composite were calculated using Materials Studio 6.0, utilizing data from the Crystallography Open Database. The molecular crystal structure simulations are shown in Figure 3. The band gaps for LiFePO_4_/C prepared using ethylene glycol, PEG4000, PVA-124, and citric acid were 0.589 eV, 0.598 eV, 0.586 eV, and 0.586 eV, respectively. While the experimental samples show slight deviations from theoretical values, these deviations are not significant. LiFePO_4_ prepared with PVA-124 and citric acid as carbon sources exhibits smaller energy gaps, indicating the potential for higher conductivity. Dragana Jugovića et al. [42] believed that smaller lattice parameters indicate that smaller LiFePO_4_ particles could be relatively easily produced, which was conducive to the integration with conductive carbon. Similarly, Huisheng Huang et al. [44] demonstrated the use of Material Studio and other software to simulate LiFePO_4_/C, aiming to understand the band gap and density of states of the energy band structure of LiFePO_4_. LiFePO_4_ usually has low conductivity; Chen et al. [45] prepared LiFePO_4_/C and found that introducing carbon can improve the conductivity of LiFePO_4_, thereby enhancing its charge discharge power and other properties.

**Figure 4:**
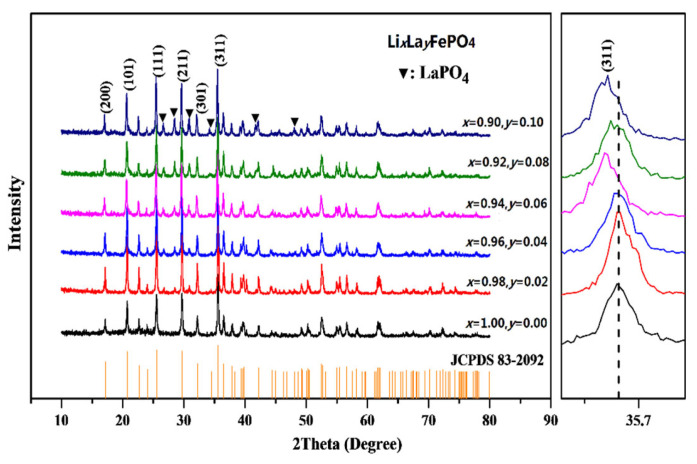
XRD pattern of Li*_x_*La*_y_*FePO_4_ (*x* = 0.9~1.0, *y* = 0~0.10) after being calcined at 700 °C for 10 h.

As shown in Figure 4, the main phase of all the synthesized samples is LiFePO_4_ [45], and all the obtained doped samples exhibit an orthorhombic crystal system belonging to the Pnma space group with an olivine structure. The main diffraction peaks (200, 101, 111, 211, 301, and 311) of all the samples align closely with those in the standard PDF card (JCPDS No. 83-2092) [6]. Table 2 shows the XRD parameters of Li*_x_*La*_y_*FePO_4_ after being calcined at 700 °C for 10 h. No impurity phases were detected in any of the samples (*x* = 1.0, *y* = 0). However, with the doping of the La ions, several impurity peaks appeared at 2θ at approximately 26.7°, 28.5°, 30.8°, 34.3°, and 41.8°. These impurity phases of LaPO_4_ can be analyzed using Jade software, and are likely due to the incomplete incorporation of La ions into the lattice, resulting in side reactions [28,29]. The shift trend of the (311) peak on the right side of Figure 4 indicates that the (311) diffraction peak tends to shift to the left due to La-ions doping [46]. Hou et al. [47] prepared LiFePO_4_@C composite and improved the charge discharge performance and low-temperature characteristics of LiFePO_4_ through magnetization treatment. Due to the larger radius of La compared to Li, the crystal structure of the doped sample is distorted and increased. Cong J et al. [25] prepared LiFe_0.98_La_0.02_O_4_/C composite and, using an EDS analysis, demonstrated that the distribution of each element in the composite material was uniform. The crystal cell volume of La doped samples was larger than that of undoped samples, further inferring that La doping has entered the LiFePO_4_ structure [26,30].

**Figure 5:**
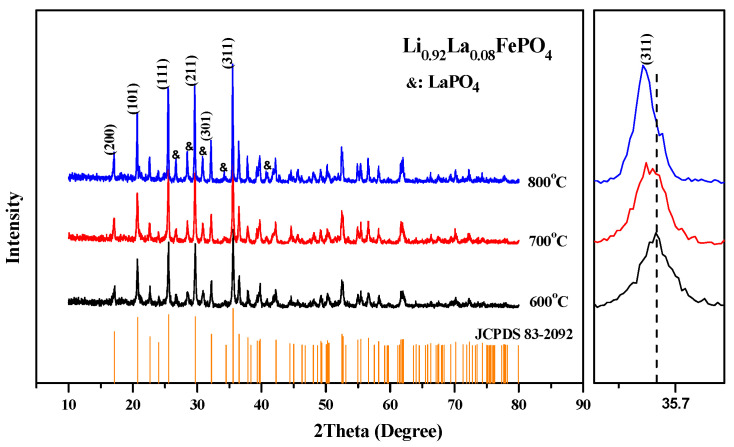
XRD pattern of Li_0.92_La_0.08_FePO_4_ after being calcined at different temperatures for 10 h.

**Figure 6:**
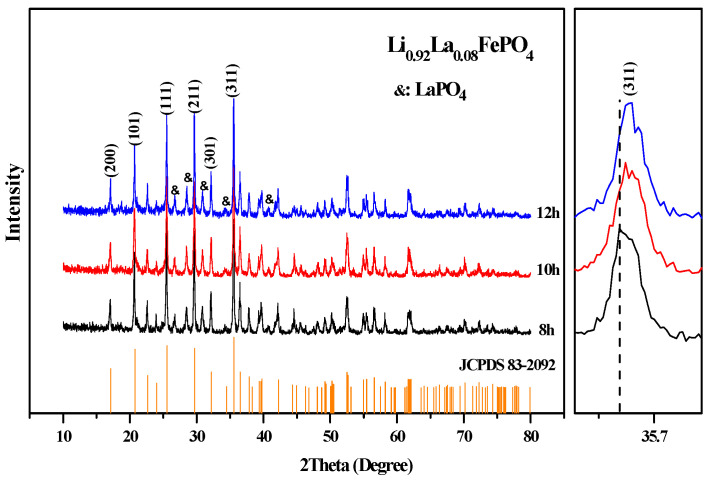
XRD pattern of Li_0.92_La_0.08_FePO_4_ after being calcined at 700 °C for different durations.

It can be seen from Table 2 that as the doping ratio increases, the sample’s cell volume first increases and then decreases, which is consistent with the initial results analyzed using the Bragg equation [46]. Figure 5 and Figure 6 illustrate the effects of different calcination temperatures and durations on the crystal structure of the samples. The main diffraction peaks (200, 101, 111, 211, 301, and 311) of Li_0.92_La_0.08_FePO_4_ are generally consistent with the findings of J.B. Goodenough et al. [1]. However, the diffraction peaks of LaPO_4_ persist at 2θ at approximately 26.7°, 28.5°, 30.8°, 34.3°, and 41.8°, which can be attributed to the previously mentioned reason [28].

According to the Bragg equation [46,47], the cell volume initially increased. Due to the larger radius of La compared to Li, the crystal structure of the doped sample will undergo distortion and increase. The crystal cell volume of La doped samples was larger than that of undoped samples, further inferring that La doping has entered the LiFePO_4_ structure. The XRD data in this article are compared with the XRD patterns of Li_1-x_La_x_FePO_4_ by Shaohua L et al. [30]. The conclusion of the XRD analysis is consistent, indicating that La doping has entered the LiFePO_4_ structure. Ji Z et al. [24] prepared LiFe_0.98_La_0.02_O_4_/C composite and, using an ICP analysis, F- was doped into the lattice of La–F-LiFePO_4_ with success; the result corresponded with the analysis of XRD.

### 2.3. Infrared Spectrum Analysis

**Figure 7:**
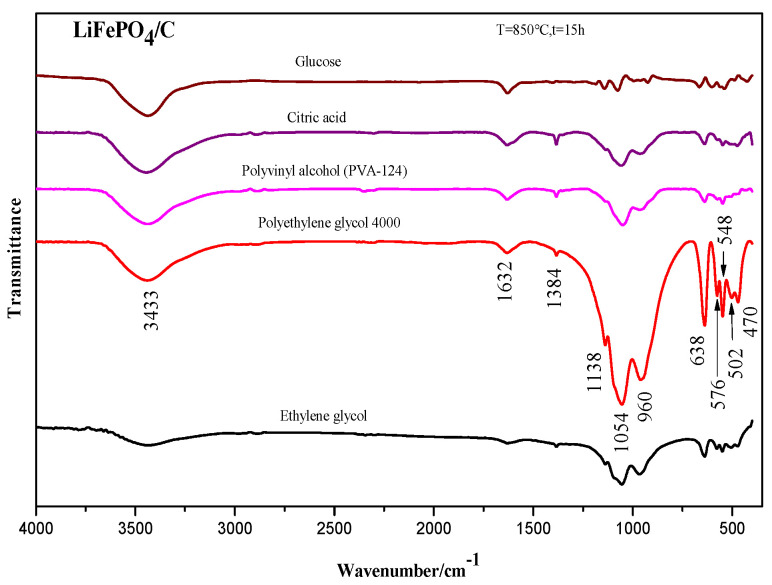
Infrared spectrum of LiFePO_4_/C composites.

To fully understand the infrared vibration absorption peaks of certain chemical bonds in the LiFePO_4_/C composites that were prepared from various carbon sources, the composites were tested and analyzed by Fourier transform infrared (FTIR) spectroscopy. The test results are shown in Figure 7. The analysis indicated that the main wave numbers of the five samples were approximately 3433 cm^−1^, 1632 cm^−1^, 1384 cm^−1^, 1138 cm^−1^, 1054 cm^−1^, 960 cm^−1^, 638 cm^−1^, 576 cm^−1^, 548 cm^−1^, 502 cm^−1^, and 470 cm^−1^.

The vibration peaks [48] of the PO_4_^3-^ ion in LiFePO_4_ primarily include 1082 cm^−1^, 980 cm^−1^, 515 cm^−1^, and 363 cm^−1^. The wave number ranges from 400 to 1200 cm^−1^ and mainly includes the infrared vibration absorption peaks of the P–O bond and the O–P–O bond [48]. The infrared vibration peaks of the Li–O bond are also present within this wave number range [49]. The group vibration modes of materials include symmetrical stretching vibration (*v*_1_), antisymmetric stretching vibration (*v*_3_), symmetrical bending vibration (*v*_2_), and antisymmetric bending vibration (*v*_4_) [50]. In LiFePO_4_, the P–O bond primarily corresponds to *v*_1_ and *v*_3_, while the O–P–O bond corresponds to *v*_2_ and *v*4 [51]. It is evident that the infrared spectra of the five samples show absorption peaks at wave numbers of 1138 cm^−1^ and 1054 cm^−1^, which are primarily attributed to *v*_3_ of the P–O bond, whereas the absorption peaks at of 960 cm^−1^ are mainly due to *v*_1_ of the P–O bond. Additionally, the absorption peaks around 638 cm^−1^, 576 cm^−1^, 548 cm^−1^, 502 cm^−1^, and 470 cm^−1^ are mainly caused by the mixed vibration of the *v*_2_ and *v*_4_ of the O–P–O bond [52]. In summary, the wave number ranges from approximately 372 to 1139 cm^−1^, primarily corresponding to the internal vibration mode of the PO_4_^3-^ ion [53]. Generally, the infrared vibrational absorption peaks of the five samples are similar to the FTIR vibrational absorption peaks of the LiFePO_4_ prepared by Abdul Halim et al. [54], which were observed at around 3400 cm^−1^, 1647 cm^−1^, 1095 cm^−1^, 984 cm^−1^, 630 cm^−1^, and 570 cm^−1^. By comparing the infrared absorption peaks of the five samples, it can be found that different carbon sources affect the LiFePO_4_/C composites differently, particularly with the wave number shifts at around 3400 cm^−1^. The absorption peak at 1138 cm^−1^ weakens when citric acid and PVA-124 are used as carbon sources, while the absorption peak at 1384 cm^−1^ weakens when glucose is used as the carbon source. The infrared vibration absorption peaks of the samples that were prepared with ethylene glycol and PEG4000 as carbon sources are relatively prominent.

**Figure 8:**
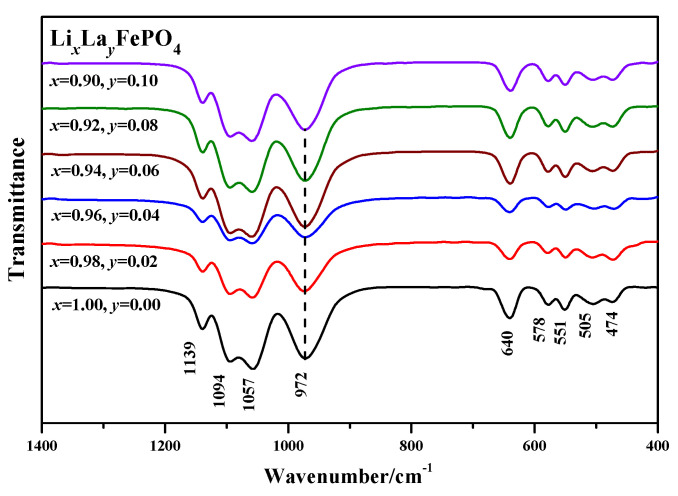
Infrared spectra pattern of Li*_x_*La*_y_*FePO_4_.

Figure 8 shows the infrared spectra pattern of Li*_x_*La*_y_*FePO_4_ after being calcined at 700 °C for 10 h. The O–P–O bond is weaker, primarily ranging between 400 and 700 cm^−1^. In the wavenumber range between 400 and 1200 cm^−1^, there is a strong absorption spectrum band from 900 to 1200 cm^−1^ and a moderately strong absorption spectrum band from 400 to 700 cm^−1^. The positions of the infrared absorption peaks for all samples are consistent with the literature [55,56]. 

Therefore, from Figure 8, it can be concluded that the absorption peaks at 1139 cm^−1^, 1094 cm^−1^, and 1057 cm^−1^ corresponded to *v*_1_, while the absorption peaks at 972 cm^−1^ corresponded to *v*_3_ [57]. In the low wavenumber region, the two absorption peaks easily overlap [58], making it difficult to clearly distinguish the vibrations.

### 2.4. Scanning Electron Microscopy Analysis

**Figure 9:**
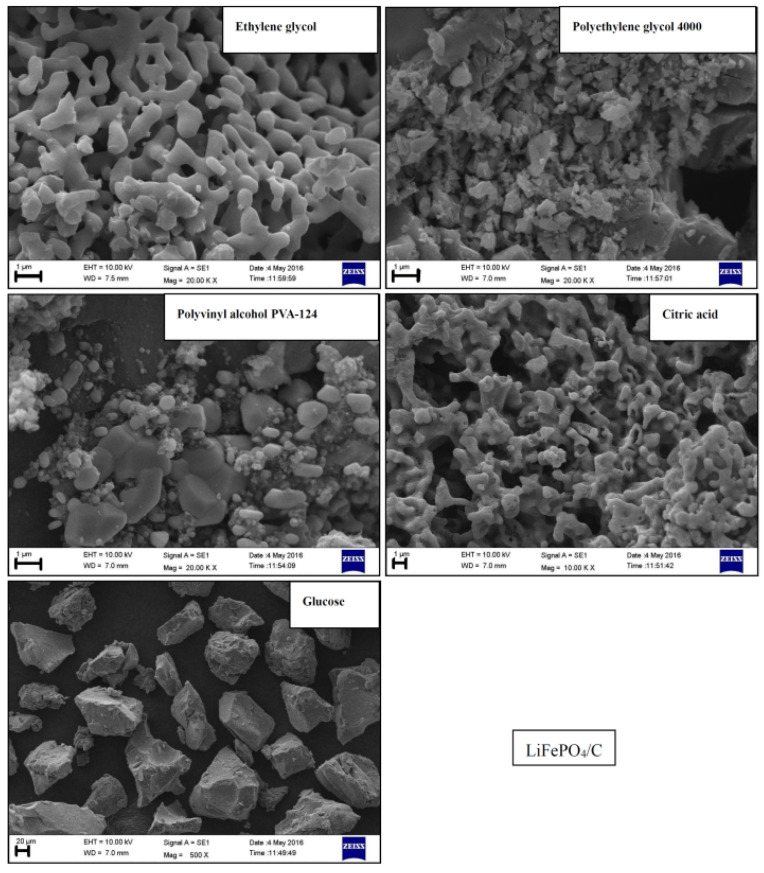
SEM of LiFePO_4_/c composites.

Scanning electron microscopy (SEM) is a crucial analytical technique for examining the microscopic surface morphology, particle size, and particle dispersion of materials [59]. To study the microscopic morphology and particle size of the LiFePO_4_/C composites that were synthesized via the sol-gel method from five different carbon sources, an SEM analysis was conducted on the five samples. The results are shown in Figure 9.

**Figure 10:**
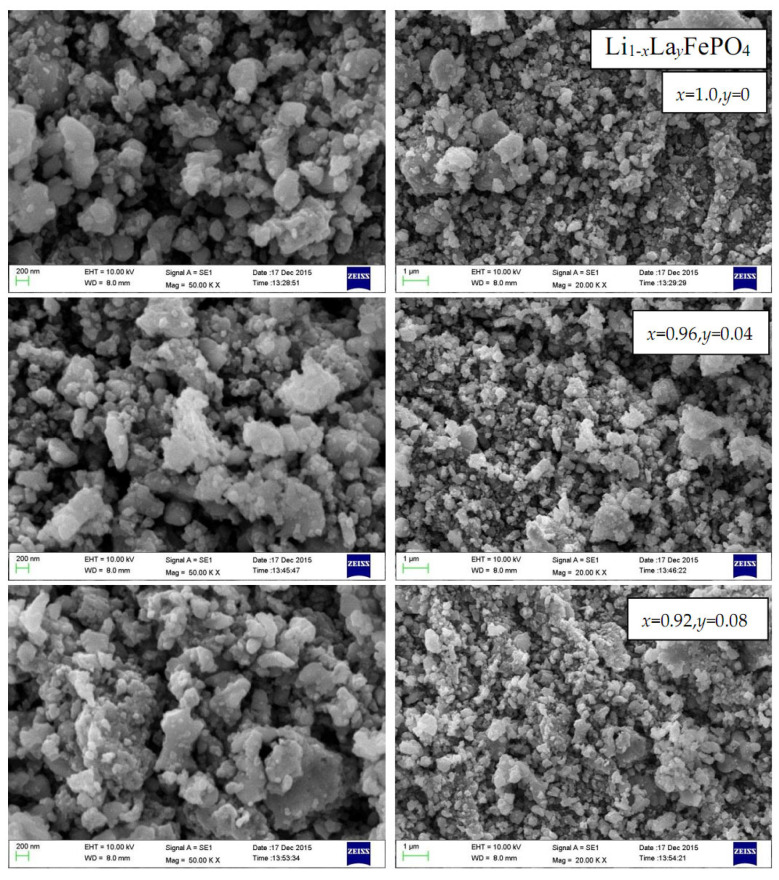
SEM of Li*_x_*La*_y_*FePO_4_ (*x* = 1.0, *y* = 0; *x* = 0.96, *y* = 0.04; *x* = 0.92, *y* = 0.08) after being calcined at 700 °C for 10 h.

The SEM image of the sample using ethylene glycol as the carbon source reveals a microstructure resembling a porous network. When combined with a conductive agent and other additives in the electrode, this network structure is conducive to improving the material’s electronic conductivity [60]. Conversely, the SEM image of the sample using PEG4000 as the carbon source reveals a loose structure with no distinct particles, similar to the LiFePO_4_ composite studied by Ercan Avci [61]. According to the microscopic morphology of the sample using PVA-124 as the carbon source, it primarily consists of two types of particles. These particles are well-connected, which helps improve the material’s electronic conductivity [62]. The microscopic morphology of the sample using citric acid as the carbon source appears relatively uniform overall, with some fractured particles and small holes visible. The SEM images of the samples using glucose as the carbon source reveal clear, dispersed, and uniformly micron-sized particle structures. Some particles exhibit a crystalline shape and appearance similar to an olivine structure. SEM [59], a crucial characterization method for materials, was used to analyze the microscopic morphology of Li*_x_*La*_y_*FePO_4_. Figure 10 shows the SEM images of Li*_x_*La*_y_*FePO_4_ (*x* = 1.0, *y* = 0; *x* = 0.96, *y* = 0.04; *x* = 0.92, *y* = 0.08) after being calcined at 700 °C for 10 h. The particles are irregularly shaped with a consistent size. However, there is relatively severe agglomeration, possibly due to increased chemical stress between the crystals, which in turn causes shrinkage and subsequent agglomeration [63,64].

### 2.5. Magnetic Analysis

**Figure 11:**
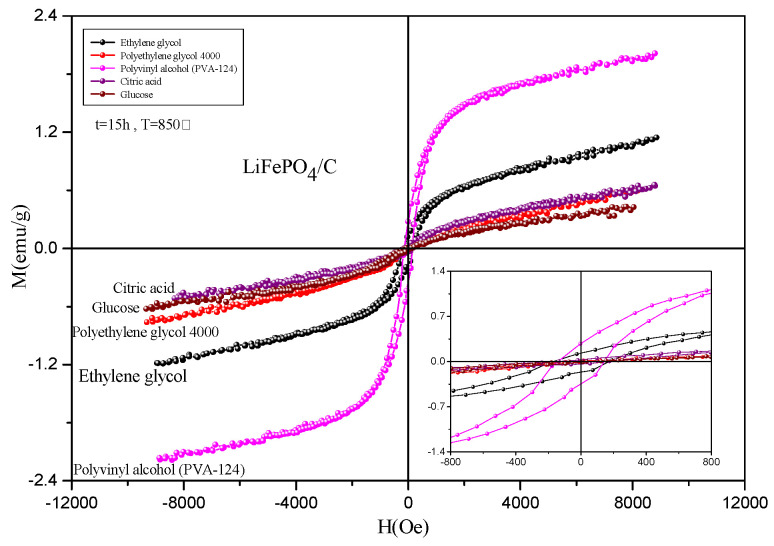
Hysteresis loop of LiFePO_4_/C composites.

**Figure 12:**
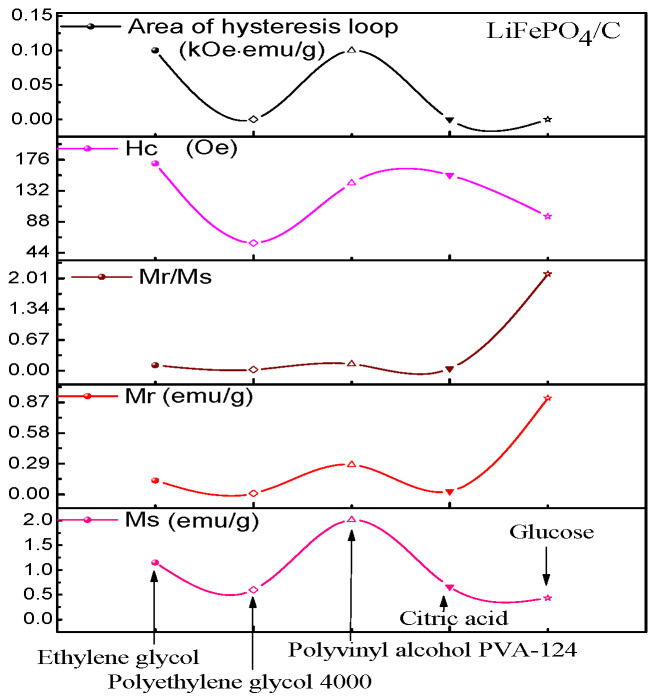
Variation trend of magnetic parameters of LiFePO_4_/C composites.

To comprehensively study the magnetic properties of the LiFePO_4_/C composites that were prepared from different carbon sources, a VSM-100 vibrating sample magnetometer (VSM) from Yingpu Magnetoelectric Company was used. The magnetic field range was set to ±8000 Oe, with a fieldincreasing step of 4 Oe. The hysteresis loops of the LiFePO_4_/C composites that were synthesized from five different carbon sources were tested at room temperature, and the results are presented in Figure 11 and Figure 12, and in Table 3. The hysteresis loop obtained from the test is similar to that of the LiFePO_4_/C composites prepared by Ming Chen et al. [65] at room temperature. The introduction of carbon significantly impacts the magnetism of LiFePO_4_. In their study, by controlling the amount of carbon, the saturation magnetization of LiFePO_4_ varied from Ms = 8.538 emu/g to Ms = 0.326 emu/g, and even to Ms = 0.313 emu/g. In this study, testing the hysteresis loops and statistically analyzing the data of the samples that were prepared with different carbon sources revealed that different carbon sources significantly influence the saturation magnetization of LiFePO_4_/C composites. The samples using PVA-124 as the carbon source exhibit the highest saturation magnetization (Ms), reaching 2.01 emu/g. In contrast, the samples using ethylene glycol as the carbon source show the highest coercivity, measured at 170.67 Oe. The hysteresis loop areas for all five samples are minimal and approach zero. The sample using glucose as the carbon source has the highest residual magnetization (Mr), recorded at 0.91 emu/g, and also the highest Mr/Ms ratio, at 2.107. For LiFePO_4_, the Neél temperature *T*_N_ is 50 K [66]. Below this temperature, the material exhibits antiferromagnetic properties, while above 50 K, it becomes paramagnetic. The composite samples that were prepared using five different carbon sources were tested with VSM at room temperature, and thus should display paramagnetic behavior.

The test results shown in Figure 11 indicate that the hysteresis loop area of the sample is almost zero, but it exhibits relatively large coercivity, with a minimum of 58.03 Oe, indicating weak ferromagnetism. This behavior may be attributed to the presence of ferrous or ferromagnetic impurities in the sample [65,67]. However, no impurity phase was found in the previous XRD test, indicating that ferromagnetic impurities might be present in very small amounts and could be encapsulated by amorphous carbon or LiFePO_4_, or their XRD diffraction peak intensity might be too weak for its XRD characteristic peak to be easily identified. This is consistent with the findings of Ming Chen et al. [65], who reported no obvious impurity peaks in the XRD patterns of some LiFePO_4_/C composite samples, yet the hysteresis loop at room temperature showed weak ferromagnetism. Hu et al. [47] prepared Li_x_FePO_4_ composite and used doped transition metal ions to alter the structure and magnetic properties of the material.

**Figure 13:**
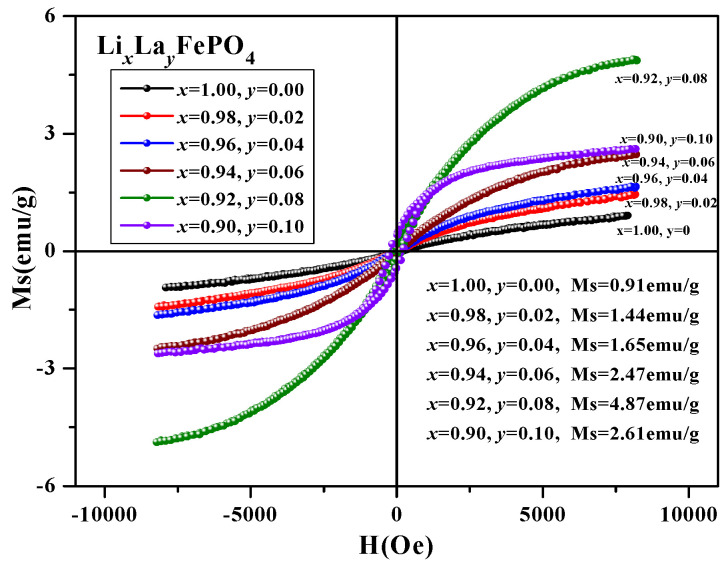
Hysteresis loop of Li*_x_*La*_y_*FePO_4_.

**Figure 14:**
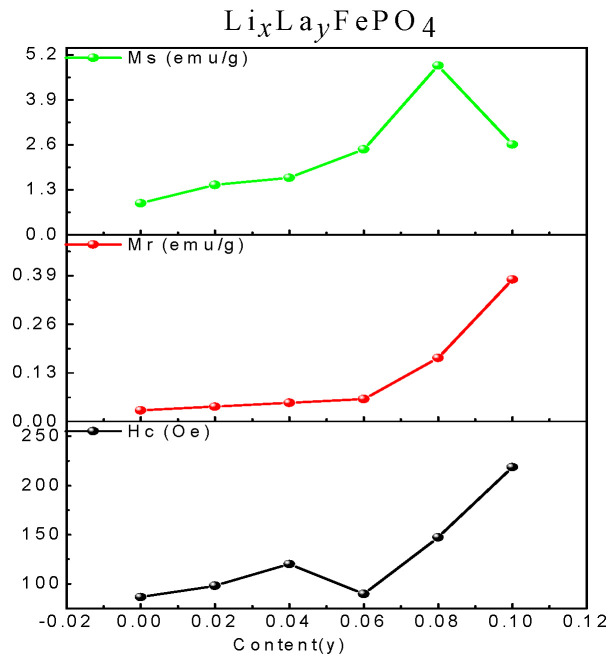
Trend of changes in magnetic parameters of Li*_x_*La*_y_*FePO_4_.

Figure 13 shows the hysteresis loop of Li*_x_*La*_y_*FePO_4_. The hysteresis loop for Li*_x_*La*_y_*FePO_4_ (*x* = 0.9~1.0, *y* = 0~0.10) after being calcined at 700 °C for 10 indicates that as the La-ion doping amount increased, the Ms of the samples first increased and then decreased, and the maximum Ms value was 4.87 emu/g (*x* = 0.92, *y* = 0.08). The magnetization of all the samples was substantially saturated. In addition, with increased La-ion doping, the Ms first increased and then decreased, with the sample at *x* = 0.92, *y* = 0.08 exhibiting the highest Ms [68,69,70,71]. Table 4 presents the magnetic parameters, and Figure 14 shows the trend of the changes in these parameters.

**Figure 15:**
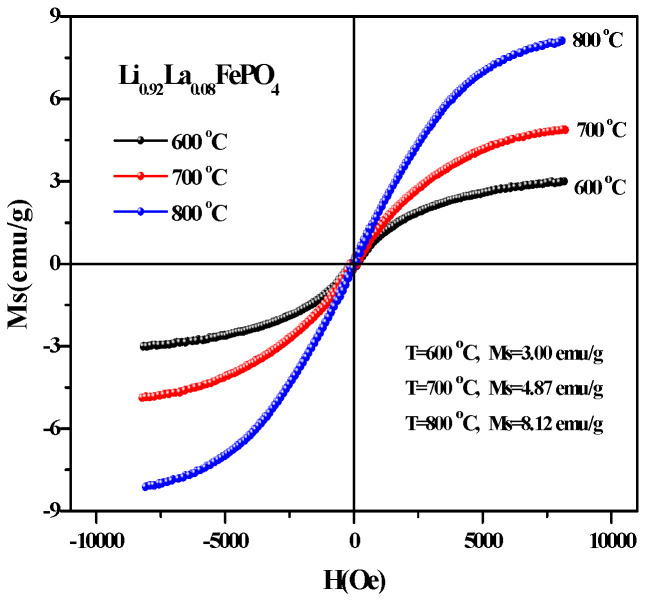
Hysteresis loop of Li_0.92_La_0.08_FePO_4_ after being calcined at different temperatures for 10 h.

**Figure 16:**
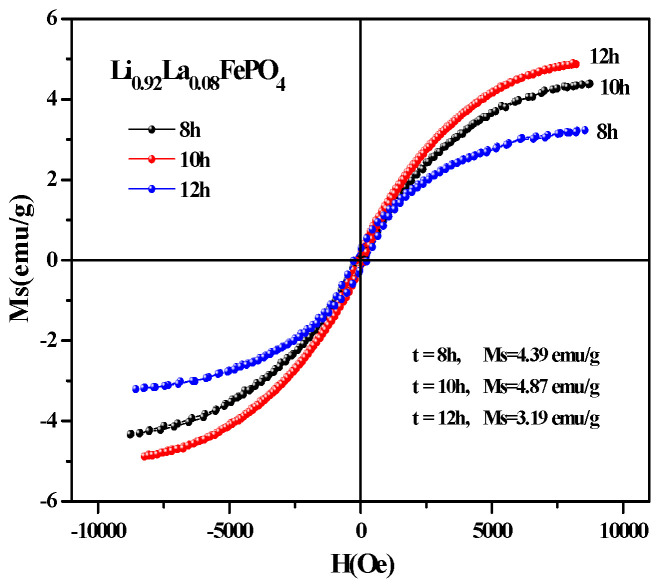
Hysteresis loop of Li_0.92_La_0.08_FePO_4_ after being calcined at 700 °C for different durations.

Figure 15 shows the hysteresis loop of Li_0.92_La_0.08_FePO_4_ after being calcined at 600 °C, 700 °C, or 800 °C for 10 h. It can be seen that the magnetization of all three samples was substantially saturated. As the calcination temperature increased, Ms also gradually increased. Table 5 indicates that the Ms values for the samples that were calcined at 600 °C, 700 °C, or 800 °C were 2.69 emu/g, 4.89 emu/g, and 7.26 emu/g, respectively. Additionally, the coercivity values shown in Table 4 reveal that the coercivity for the samples that were calcined at 600 °C, 700 °C, or 800 °C were 185.24 Oe, 147.39 Oe, and 58.21Oe, respectively, indicating weak ferromagnetic properties. This weak ferromagnetism may be attributed to ferromagnetic impurities [72]. Figure 16 shows the hysteresis loop of Li_0.92_La_0.08_FePO_4_ after being calcined at 700 °C for different durations. As the calcination time increased, Ms gradually increased. The hysteresis loop of Li_0.92_La_0.08_FePO_4_ after being calcined at 700 °C for different durations indicates that the maximum Ms value was 4.87 emu/g (calcination time 10 h) and the minimum Ms value was 3.19 emu/g (calcination time 12 h). The magnetic properties of LiFePO_4_ are closely related to the electron spin and distribution in its structure, which affect the conductivity of LiFePO_4_. Zhou et al. [73] prepared Li_x_FePO_4_ composite and using doped transition metal ions to alter the magnetic orientation. The direction of the magnetic moment is closely related to the deintercalation of lithium in LiFePO_4_.

### 2.6. Mössbauer Spectra Analysis

**Figure 17:**
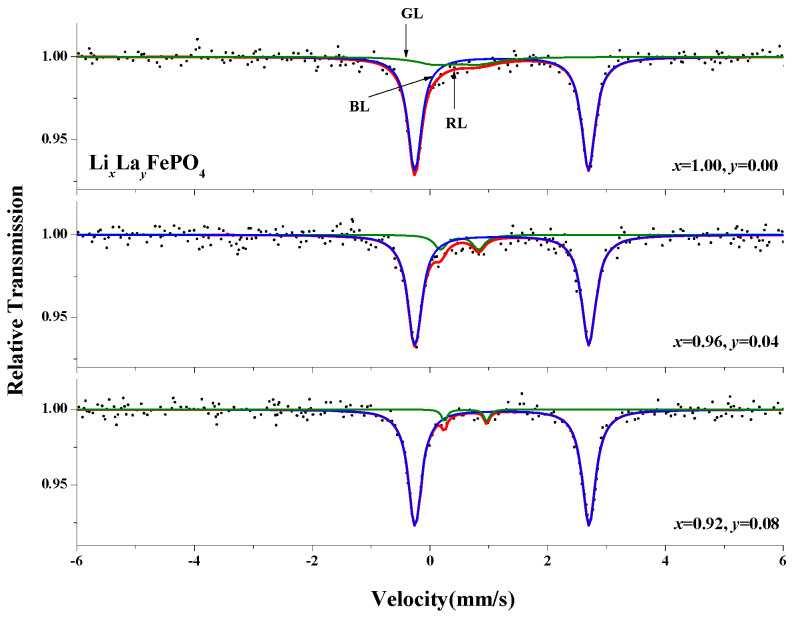
Mössbauer spectra of Li*_x_*La*_y_*FePO_4_ (*x* = 1.00, *y* = 0; *x* = 0.96, *y* = 0.04; *x* = 0.92, *y* = 0.08) after being calcined at 700 °C for 10 h.

The Mössbauer spectra of Li*_x_*La*_y_*FePO_4_ (*x* = 1.00, *y* = 0; *x* = 0.96, *y* = 0.04; *x* = 0.92, *y* = 0.08) were measured at room temperature, and the original data were fitted using Mosswinn3.0 software. Figure 17 shows the Mössbauer spectra of Li*_x_*La*_y_*FePO_4_. The final results were obtained by fitting the original data with two paramagnetic doublets. As shown in Table 6, the isomer shifts (ISs) of Doublet(1) for the three samples were 1.218 mm/s, 1.220 mm/s, and 1.224 mm/s, respectively, indicating the presence of Fe^2^⁺ compounds. In addition, A.A.M. Prince et al. [74] and Dominika Baster et al. [75] found that the quadrupole splitting (QS) of Doublet(1) was significant. As shown in Table 6, the proportions of Doublet(1) for the three samples were 85.5%, 89.9%, and 96.0%, respectively, while the proportions of Doublet(2) were 14.5%, 10.1%, and 4.0%, respectively. Therefore, the main component in the samples was the Fe^2^⁺ compound, with a certain proportion of the Fe^3^⁺ compound [76].

## 3. Experimental Section

**Figure 18:**
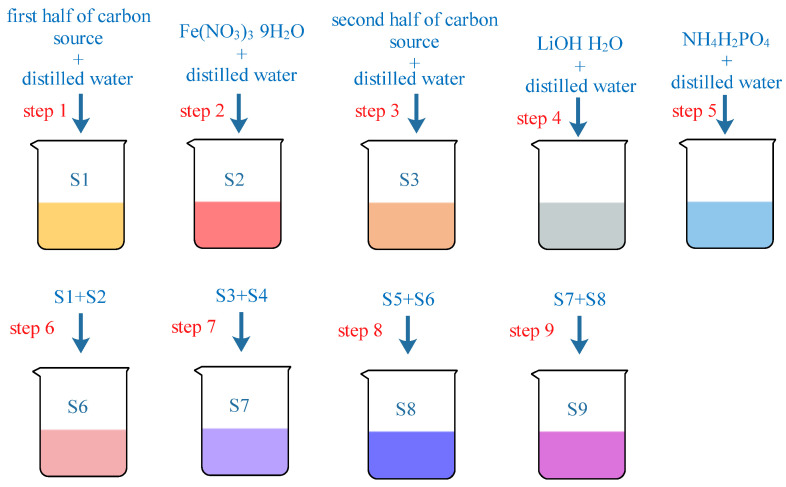
Dissolution sequence of pharmaceutical reagents for preparation of LiFePO_4_/C.

**Figure 19:**
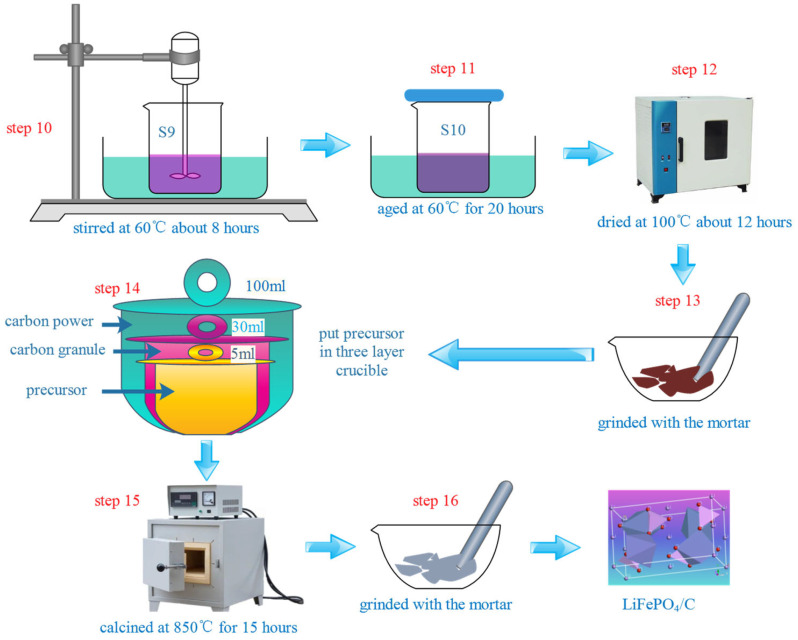
Preparation process of LiFePO_4_/C.

**Figure 20:**
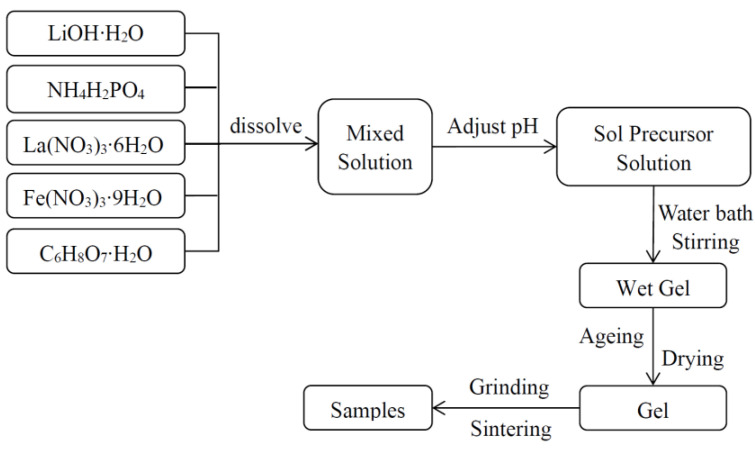
Flow chart of the preparation process.

The LiFePO_4_/C composites were prepared using the sol-gel method. This study focused on the effects of different carbon sources on the phase crystal structure, particle morphology, and magnetic properties of the LiFePO_4_/C composites. The chemical reagents used for their preparation included various carbon sources (C_2_H_6_O_2_, C_2_H_6_O_2_, HO(CH_2_CH_2_O)*_n_*H, [CH_2_CH(OH)]*_n_*, C_6_H_8_O_7_·H_2_O, and C_6_H_12_O_6_·H_2_O), a phosphorus source (NH_4_H_2_PO_4_), an iron source (Fe(NO_3_)_3_·9H_2_O), and a lithium source (LiOH·H_2_O). All of the reagents were purchased from Xilong Science Co., Ltd. The molar ratio of the carbon sources, phosphorus source, iron source, and lithium source was 2:1:1:1. HO(CH_2_CH_2_O)*_n_*H and [CH_2_CH(OH)]*_n_* were weighed to match the mass of C_2_H_6_O_2_. The dissolution sequence of the chemical reagents for the preparation of the LiFePO_4_/C is shown in Figure 18. Preparation process of LiFePO_4_/C was showed in Figure 19. Li*_x_*La*_y_*FePO_4_ (*x* = 0.9~1.0, *y* = 0~0.1) materials were prepared by the sol-gel method with citric acid as a complexing agent to investigate the impact of different La^2+^ ratio, calcination temperature, and calcination time. The flow chart of the process is shown in Figure 20. La(NO_3_)_3_·6H_2_O was purchased from Tianjin Guangfu Fine Chemical Research Institute.

Specific implementation steps of preparation of Li*_x_*La*_y_*FePO_4_ are:

Step 1: Calculate the raw materials of Li*_x_*La*_y_*FePO_4_ (*x* = 1.0, *y* = 0; *x* = 0.98, *y* = 0.02; *x* = 0.96, *y* = 0.04; *x* = 0.94, *y* = 0.06; *x* = 0.92, *y* = 0.08; *x* = 0.90, *y* = 0.10) according to the stoichiometric ratio, and then weigh the raw materials into small beakers, numbered as A_1_–A_6_, according to the calculated amount;

Step 2: Add about 100 mL of deionized water to the A_1_ to A_6_ small beakers and stir continuously to obtain a red wine clear liquid of the A_1_ to A_6_ solutions after dissolving entirely;

Step 3: Transfer the aqueous solutions A_1_ to A_6_ to the fume hood, then add ammonia into the aqueous solution until the pH value is 9 while stirring continuously, and subsequently obtain about 100 to 120 mL of burgundy clear Sol precursor solution B_1_ to B_6_;

Step 4: Continuously stir the precursor solutions B_1_ to B_6_ in a water bath at 80 °C for about 3 h, and stop when a wet gel is formed; 

Step 5: Dry the wet gel at 120 °C for 12 h in an air-drying oven after the wet gel is aged at 80 °C for 12 h;

Step 6: Grind the dry gel with an agate mortar before putting 5 mL of dry gel powder or smaller pieces of dry gel into a lidded porcelain crucible;

Step 7: Introduce nitrogen for about 40 min to remove the air in the quartz tube after transferring the dry gel powder from the porcelain crucible to the tube furnace, and then calcine and cool to room temperature in a nitrogen atmosphere;

Step 8: Put the loose-shaped samples from the porcelain crucible to the agate mortar, then finely grind it again with the agate mortar to obtain the final samples.

The various analytical techniques (TG-DTA, XRD, SEM, Mössbauer, VSM) were used to determine the following features: the impact of different doping amounts of La^2+^ ion, calcination temperature, and calcination time on the structure, functional groups, chemical bonding, particle shape and size, magnetic performance, and hyperfine interaction of samples

## 4. Conclusions

In this study, five different carbon sources, including ethylene glycol (C_2_H_6_O_2_, analytical grade AR), polyethylene glycol 4000 (PEG4000; HO(CH_2_CH_2_O)*_n_*H, chemically pure CP), polyvinyl alcohol (PVA-124; [CH_2_CH(OH)]*_n_*, analytical grade AR), citric acid (C_6_H_8_O_7_·H_2_O, analytical grade AR), and glucose (C_6_H_12_O_6_·H_2_O, analytical grade AR), were used, and LiFePO_4_/C composites were synthesized using a one-step sol-gel method. The crystal phase structure, functional groups, chemical bonds, microscopic surface morphology, and magnetic properties of the LiFePO₄/C composites were analyzed using XRD, FTIR, SEM, and VSM. The results indicate that the LiFePO₄/C composites that were prepared with these five carbon sources exhibit the complete standard peaks characteristic of pure LiFePO₄. The introduction of different carbon sources results in shifts in certain infrared characteristic peaks. These five carbon sources significantly affect the microstructure and magnetic properties of the composites. The sample using ethylene glycol as the carbon source forms a porous network structure, which is conducive to improving electronic conductivity. However, the sample using glucose as the carbon source exhibits distinct particles with good dispersion, resembling olivine crystal morphology. The sample using PVA-124 as the carbon source shows the highest relative Ms, measured at 2.01 emu/g, whereas the sample using ethylene glycol as the carbon source has the highest coercivity, recorded at 170.67 Oe. In short, among the five samples, the LiFePO₄/C composite that was prepared using ethylene glycol as the carbon source shows better electromagnetic properties. All of the synthesized LiFePO₄ samples doped with La have an olivine structure. However, the presence of LaPO_4_ impurities from side reactions indicates that the La ions were only partially doped into the lattice. The IR analysis indicates that all samples show characteristic infrared absorption peaks; the magnetic analysis suggests that the observed weak ferromagnetism may be due to the presence of weak ferromagnetic impurities; the Mössbauer spectroscopy indicates that Fe^2+^ compounds are the main components in the samples, accompanied by some Fe^3^⁺ compounds, indicating the coexistence of Fe^3^⁺/Fe^2^⁺ valence states. The Mössbauer spectra of Li*_x_*La*_y_*FePO_4_ (*x* = 1.00, *y* = 0; *x* = 0.96, *y* = 0.04; *x* = 0.92, *y* = 0.08) after being calcined at 700 °C for 10 h indicate that all samples contain Doublet(1) and Doublet(2) peaks, dominated by Fe^2+^ compounds. The proportions of Fe^2+^ are 85.5% (*x* = 1.0, *y* = 0), 89.9% (*x* = 0.96, *y* = 0.04), and 96.0% (*x* = 0.92, *y* = 0.08). The maximum IS and QS of Doublet(1) for the three samples are 1.224 mm/s and 2.956 mm/s, respectively.

## Figures and Tables

**Table 1:** XRD parameters of LiFePO_4_/C composites.

Samples	Carbon Source	Average Lattice Constants		
		a-Axis (Å)	b-Axis (Å)	c-Axis (Å)
PDF#83-2092		10.3340	6.0100	4.6930
1	Ethylene glycol	10.2769	5.9870	4.6842
2	PEG4000	10.2509	5.9877	4.6700
3	PVA-124	10.2784	5.9599	4.6906
4	Citric acid	10.2646	5.9844	4.6863
5	Glucose	10.0212	5.8209	4.7350

**Table 2:** XRD parameters of Li*_x_*La*_y_*FePO_4_.

(*x*, *y*)	Lattice Parameters				Crystallite Size (Å)
	a (Å)	b (Å)	c (Å)	Vol (Å^3^)	
(1.00, 0.00)	10.34119	6.00442	4.70433	292.10	493
(0.98, 0.02)	10.33708	6.00860	4.69580	292.21	678
(0.96, 0.04)	10.34229	6.01088	4.69574	292.52	668
(0.94, 0.06)	10.34652	6.01306	4.71540	293.36	488
(0.92, 0.08)	10.37129	6.01088	4.69703	292.82	407
(0.90, 0.10)	10.35730	6.02266	4.70102	293.24	497

**Table 3:** Magnetic parameters of LiFePO_4_/C composites.

Samples	1	2	3	4	5
Carbon source	Ethylene glycol	PEG4000	PVA-124	Citric acid	Glucose
Ms (emu/g)	1.15	0.60	2.01	0.66	0.43
Mr (emu/g)	0.13	0.01	0.28	0.03	0.91
Mr/Ms	0.115	0.023	0.140	0.041	2.107
Hc (Oe)	170.67	58.03	142.68	154.15	95.70
Area of hysteresis (kOe·emu/g)	0.1	0.0	0.1	0.0	0.0

**Table 4:** Magnetic parameters of Li*_x_*La*_y_*FePO_4_ after being calcined at 700 °C for 10 h.

(*x*, *y*)	(1.00, 0)	(0.98, 0.02)	(0.96, 0.04)	(0.94, 0.06)	(0.92, 0.08)	(0.90, 0.10)
Ms (emu/g)	0.91	1.44	1.65	2.47	4.89	2.61
Mr (emu/g)	0.03	0.04	0.05	0.06	0.17	0.38
Hc (Oe)	86.55	98.10	120.15	89.68	147.39	218.59

**Table 5:** Magnetic parameters of Li_0.92_La_0.08_FePO_4_.

*T* (°C)	Ms (emu/g)	Mr (emu/g)	Hc (Oe)
600	2.69	0.17	185.24
700	4.89	0.17	147.39
800	7.26	0.08	58.21

**Table 6:** Mössbauer parameters of Li*_x_*La*_y_*FePO_4_.

(*x*, *y*)	Component	IS (mm/s)	QS (mm/s)	LW (mm/s)	A (%)
(1.00, 0.00)	Doublet(1)	1.218	2.951	0.285	85.5
	Doublet(2)	0.452	0.717	0.832	14.5
(0.96, 0.04)	Doublet(1)	1.220	2.956	0.297	89.9
	Doublet(2)	0.503	0.650	0.255	10.1
(0.92, 0.08)	Doublet(1)	1.224	2.956	0.282	96.0
	Doublet(2)	0.605	0.719	0.109	4.0

## Data Availability

The original contributions presented in the study are included in the article, further inquiries can be directed to the corresponding authors.
